# Therapeutic Challenges in the Use of Chemotherapy and Cyclin-Dependent Kinase 4/6 Inhibitors in a Patient With Charcot-Marie-Tooth Disease and Breast Cancer

**DOI:** 10.7759/cureus.90239

**Published:** 2025-08-16

**Authors:** Pedro Mota Araújo, Renato Cunha, Marta Sousa

**Affiliations:** 1 Oncology, Unidade Local de Saúde de Trás-os-Montes e Alto Douro, E.P.E., Vila Real, PRT

**Keywords:** breast cancer, charcot-marie-tooth disease, chemotherapy, cyclin-dependent kinase 4/6 inhibitors, neuropathy

## Abstract

We report a rare clinical scenario involving a 65-year-old female patient with Charcot-Marie-Tooth (CMT) disease who was diagnosed with early-stage breast carcinoma. The patient underwent neoadjuvant chemotherapy, followed by surgery and adjuvant endocrine therapy. During treatment, she developed grade 3 peripheral neuropathy, leading to the early discontinuation of paclitaxel and subsequent therapeutic adjustments. This report highlights the challenges of managing patients with neuromuscular comorbidities undergoing chemotherapy and the use of cyclin-dependent kinase 4/6 (CDK 4/6) inhibitors as adjuvant therapy in breast cancer treatment.

## Introduction

Charcot-Marie-Tooth (CMT) disease comprises a group of hereditary neuropathies affecting the peripheral nerves, characterized by symptoms such as muscle weakness, atrophy, and peripheral sensory alterations, particularly in the limbs [1]. CMT is classified into various subtypes, with CMT1A being the most common, resulting from a duplication of the PMP22 gene [1]. This variant typically manifests in childhood or adolescence and progresses slowly, potentially leading to foot deformities, gait difficulties, and, in advanced cases, impaired hand function [1]. This pre-existing peripheral nerve dysfunction may predispose patients to developing severe chemotherapy-induced peripheral neuropathy (CIPN), a frequent and sometimes disabling adverse effect of taxane-based regimens such as paclitaxel.

Breast cancer is the most common malignant neoplasm among women worldwide, accounting for 12% of all new cancer cases in 2020 and causing over 685,000 deaths that same year [2]. Luminal breast cancer is characterized by the positive expression of hormone receptors (HR), estrogen and/or progesterone, and the absence or low expression of human epidermal growth factor receptor 2 (HER2). According to the 2023 guidelines of the European Society for Medical Oncology (ESMO) [3], the treatment of early-stage luminal breast cancer is based on surgery, radiotherapy, and/or systemic therapy. In higher-risk clinical cases, adjuvant endocrine therapy may be combined with cyclin-dependent kinase 4/6 (CDK 4/6) inhibitors, such as abemaciclib or ribociclib, to reduce the risk of recurrence [3].

Regarding systemic treatments, one of the most commonly used chemotherapy protocols in the treatment of breast cancer is the combination of dose-dense (dd) doxorubicin (A) and cyclophosphamide (C) followed by paclitaxel (T), particularly in early-stage disease with high risk of recurrence [4]. The clinical efficacy of this protocol is well established, with significant improvements in disease-free survival and overall survival compared to conventional schedules. However, the therapeutic benefits of the ddAC-T regimen are frequently offset by its cumulative toxicities, with CIPN being among the most frequent and disabling adverse effects [5]. Clinical studies estimate that up to 70% of patients treated with paclitaxel develop some degree of neuropathy, with a substantial risk of persistent symptoms, such as paresthesia, neuropathic pain, allodynia, and, in severe cases, functional impairment that can negatively affect quality of life and treatment adherence [5].

Given the high prevalence and functional impact of CIPN, it is crucial to further investigate its characterization, prevention, and management, especially in the context of dose-dense regimens such as ddAC-T. A deeper understanding of the underlying pathophysiological mechanisms and predisposing factors could aid in devising more individualized treatment approaches, thereby minimizing toxicity without compromising oncologic efficacy.

## Case presentation

The patient was a 65-year-old retired female, formerly employed as a telephone operator, who was autonomous and lived alone, with a past medical history of CMT disease type 1A and dyslipidemia. Her surgical history included a total left hip prosthesis and multiple orthopedic procedures on all four limbs due to complications of her underlying syndrome. She was on chronic lipid-lowering therapy with rosuvastatin.

**Figure 1:**
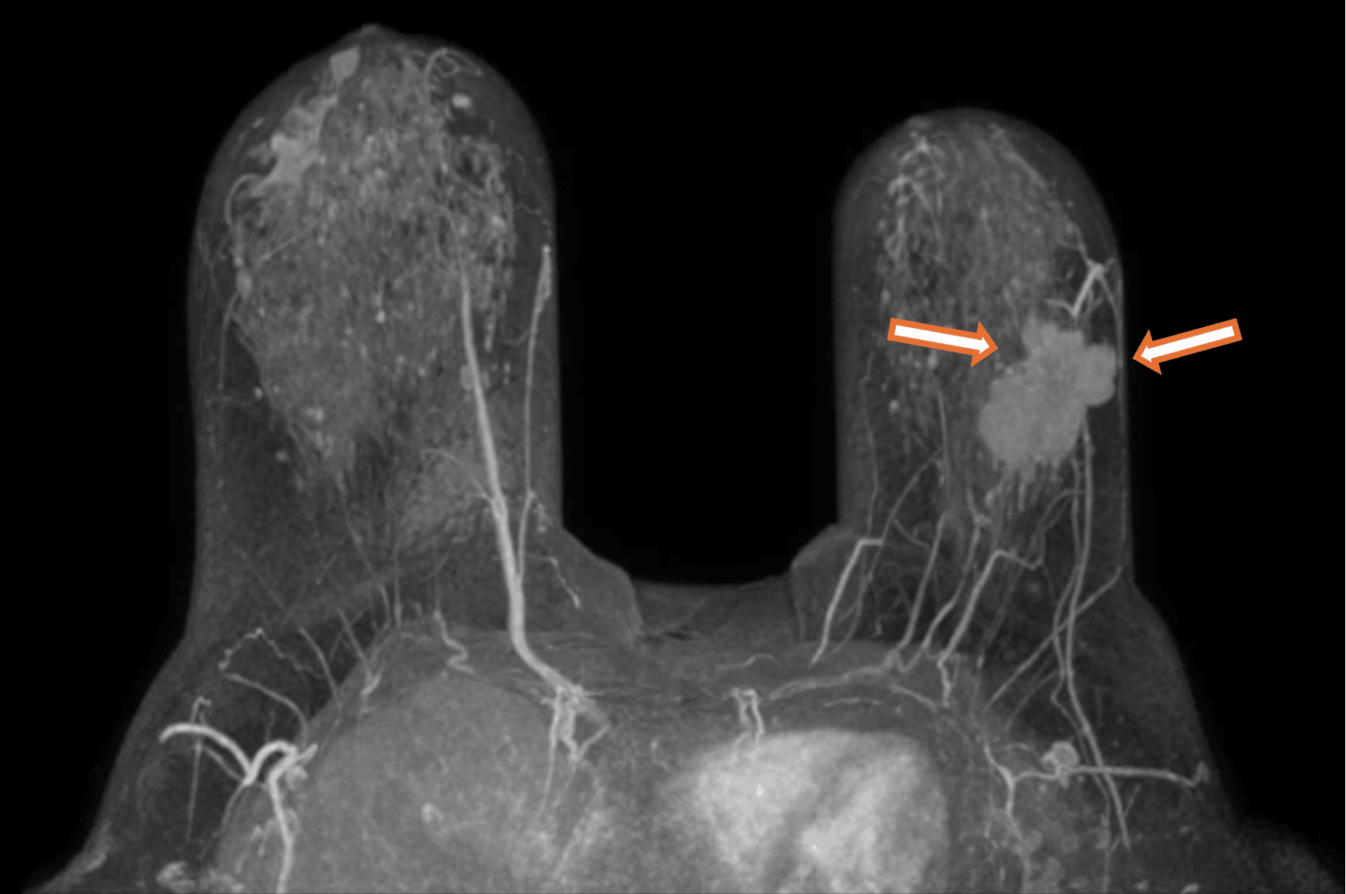
Breast MRI before neoadjuvant chemotherapy The two arrows indicate the primary tumor in the outer quadrants of the left breast MRI: magnetic resonance imaging

In November 2022, she was diagnosed with left breast carcinoma with positive hormonal receptors (estrogen receptors: 90-100%; progesterone receptors: 30-40%) and HER2-low status (immunohistochemistry score 1+) with Ki67 at 30%. Breast MRI revealed a 36 mm mass at the transition of the outer quadrants of the left breast (Figure 1), along with left axillary lymphadenopathy measuring 10 mm and smaller associated lymph nodes (LNs). A staging thoraco-abdomino-pelvic CT scan ruled out distant metastases but reported right diaphragmatic eventration/paralysis. The clinical staging was classified as cT2N+ (N2-3) M0, according to the eighth edition of the American Joint Committee on Cancer (AJCC) staging system [6].

On physical examination, the patient was in good general condition, with an Eastern Cooperative Oncology Group (ECOG) performance status of 1, attributed to peripheral motor neuropathy secondary to her syndrome. Neoadjuvant chemotherapy was proposed with a regimen of four dose-dense cycles of doxorubicin 60 mg/m² and cyclophosphamide 600 mg/m² every 14 days, followed by 12 weekly cycles of paclitaxel 80 mg/m², starting at a reduced dose due to the risk of exacerbating peripheral neuropathy, with the possibility of subsequent dose adjustments based on tolerance.

The patient completed four dose-dense cycles of doxorubicin and cyclophosphamide, experiencing asthenia and grade 1 peripheral neuropathy as adverse effects, according to the classification in the National Cancer Institute Common Terminology Criteria for Adverse Events (NCI-CTCAE) version 5.0 [7]. She was then prescribed weekly paclitaxel, receiving an initial 50% dose reduction, which was adjusted to a 30% reduction in the second cycle due to excellent tolerance. By the fourth and fifth cycles, the dose reduction was set at 20%. However, as neuropathy worsened, the sixth and seventh cycles were administered with a 30% dose reduction. By the eighth cycle, neuropathy had progressed to grade 2, necessitating a 50% dose reduction. Treatment was ultimately discontinued after eight cycles due to the development of grade 3 peripheral neuropathy. Subsequently, following the discontinuation of systemic chemotherapy treatment, the patient ultimately improved and recovered from peripheral neuropathy to her baseline level.

**Figure 2:**
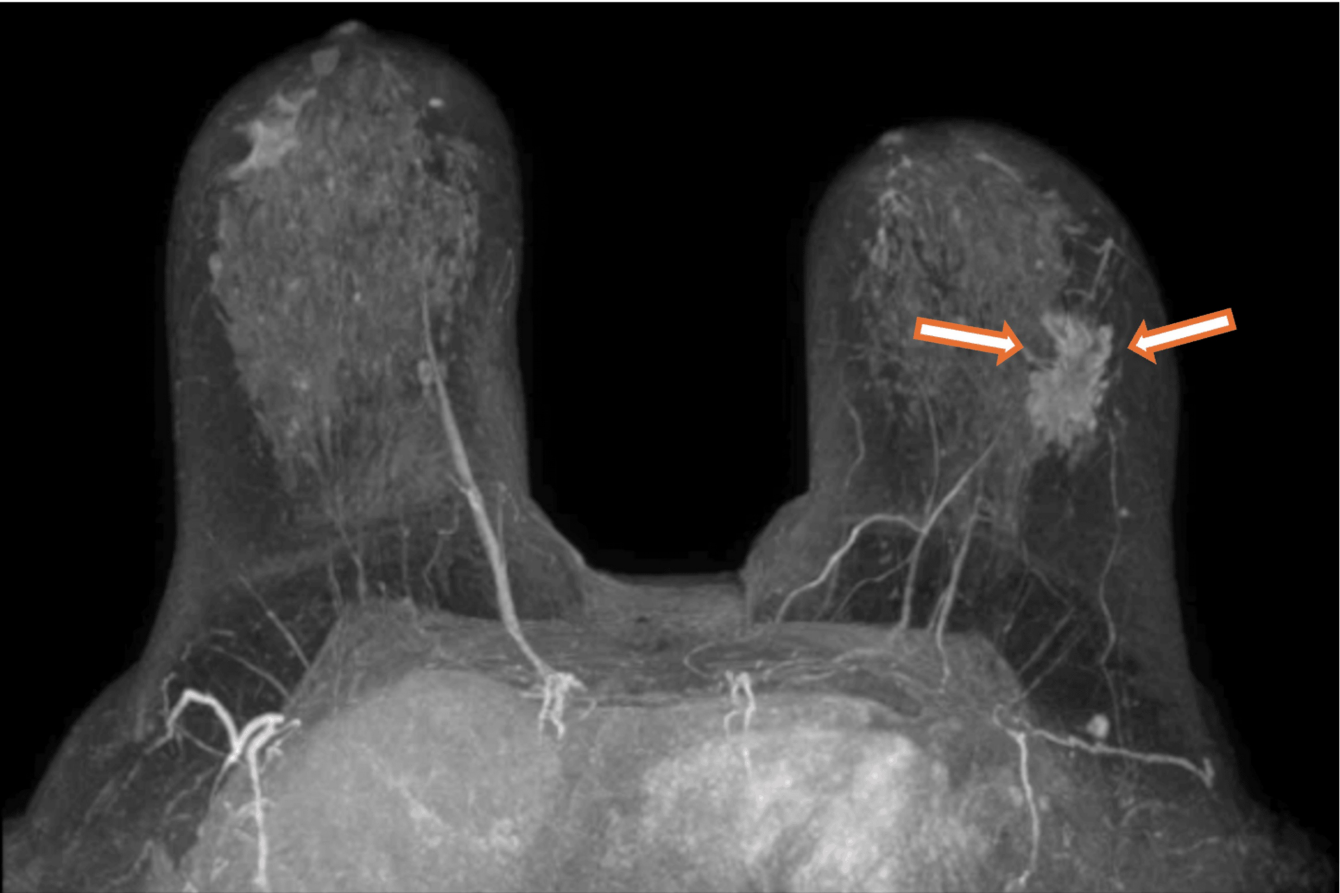
Breast MRI after neoadjuvant chemotherapy The two arrows indicate the residual tumor in the outer quadrants of the left breast, showing partial response to treatment MRI: magnetic resonance imaging

A follow-up breast MRI after neoadjuvant chemotherapy showed a reduction in tumor burden, with the primary lesion decreasing from 36 to 29 mm (Figure 2), along with a reduction in axillary LN size. In July 2023, the patient underwent a lumpectomy with lymphadenectomy. Histopathological analysis confirmed the initial diagnosis, and the disease was staged as ypT2N2a (invasive carcinoma, NST, grade 2 histology, measuring 25 mm and with five of 18 LNs positive for malignancy) according to the eighth edition of the AJCC staging system [6]. In August 2023, adjuvant endocrine therapy with letrozole was initiated in combination with the selective CDK4/6 inhibitor ribociclib (although abemaciclib was approved, its was not used due to lack of financial reimbursement; therefore, taking into account the already published results of the NATALEE trial [8], ribociclib was administered off-label, despite the lack of formal approval at the time), alongside radiotherapy. The main adverse effects of adjuvant therapy included grade 1 asthenia, nausea, and constipation, according to the classification of the NCI-CTCAE version 5.0 [7].

In January 2024, the patient was admitted to the ICU due to severe respiratory failure secondary to influenza A pneumonia. She responded well to treatment and was discharged after a one-month hospitalization. As a consequence of clinical deterioration and prolonged hospitalization, the patient experienced a loss of functional capacity, being classified with an ECOG performance status of 3. Due to this event, although not directly attributed to ribociclib toxicity, it was decided to discontinue this drug after completing only six months of treatment, while maintaining letrozole monotherapy.

In May 2024, surveillance breast ultrasound and mammography showed no evidence of oncological disease. A follow-up thoraco-abdomino-pelvic CT scan revealed sequelae-related consolidation in the left upper lobe and lingula, significant regression of ground-glass opacities, diffuse left breast densification, and a stable spiculated density in the left axilla, with no signs of malignancy. The patient remains under clinical surveillance, with an ECOG performance status of 1 and grade 1 baseline peripheral sensory and motor neuropathy.

## Discussion

CMT disease is one of the most common hereditary neuropathies, with an estimated prevalence of one in 2,500 individuals [9]. Although this disease does not significantly reduce life expectancy, it causes substantial morbidity due to the progressive motor and sensory neuropathy that characterizes the condition [1]. This is exemplified in the present case report, where the patient exhibited grade 1 motor and sensory peripheral neuropathy with functional limitations, according to the classification in the NCI-CTCAE version 5.0 [7]. The main treatment for patients with CMT disease involves physical and/or occupational therapy [10]. Additionally, orthopedic devices such as specialized footwear or leg braces can help improve balance and mobility, although, in rare cases, surgical intervention may be indicated [10].

Breast cancer is the most common malignant neoplasm among women worldwide and the leading cause of cancer-related mortality in this population [2]. The majority of newly diagnosed breast cancer cases are localized, accounting for approximately 65-70% of cases, and early detection and appropriate treatment significantly reduce mortality rates, with documented five-year survival rates exceeding 90% in developed countries [3]. Our patient presented with HR-positive, HER2-negative breast cancer, the most common breast cancer subtype, comprising 70-75% of cases [11]. Early-stage HR-positive, HER2-negative breast cancer is treated with curative intent, typically involving surgery with or without radiotherapy or chemotherapy, followed by adjuvant endocrine therapy for 5-10 years [12]. Adjuvant endocrine therapy improves clinical outcomes in these patients; however, recurrence occurs in 27-37% of those with stage II disease and 46-57% of those with stage III disease according to the eighth edition of the AJCC staging system [6], with the potential for late recurrence up to 20 years after diagnosis [13].

For most HR-positive, HER2-negative breast cancers detected through screening, surgery is the primary treatment modality. However, in cases with larger tumors or clinical nodal involvement, neoadjuvant systemic therapy may be preferred [14]. Neoadjuvant chemotherapy can be effective in surgically downstaging HR-positive, HER2-negative breast cancers; however, pathological complete response is uncommon, though more frequently observed in young patients and/or those with high-grade tumours [14]. Anthracycline-based and taxane-based therapies are frequently used as preoperative systemic treatments for patients presenting with locally advanced disease at presentation [4,15]. The sequential incorporation of a taxane following an anthracycline has been shown to improve patient outcomes in both the neoadjuvant and adjuvant settings [15].

The peripheral neuropathy, predominantly sensory, associated with paclitaxel administration is well documented in the literature and is estimated to be moderate to severe (NCI-CTCAE version 5.0 grade 2/3) in approximately 30% of cases [7,16]. In some centers, the use of neurotoxic agents, such as paclitaxel, is relatively contraindicated in patients with pre-existing neuropathy. Although peripheral neuropathy is a frequent adverse effect of paclitaxel, severe cases are rare when the recommended doses and administration schedule are followed [17]. In case of severe peripheral neuropathy, the benefits of continuing treatment should be carefully weighed against the potential risks, and, if treatment continuation is necessary, a dose reduction of paclitaxel is recommended [17].

Cases of coexisting CMT disease and breast cancer are rarely reported in the literature. The use of potentially neurotoxic agents, such as paclitaxel, raises concerns regarding their adverse effect profile, particularly in these patients. The major side effects associated with paclitaxel include myelosuppression, peripheral neuropathy, hypersensitivity reactions, and alopecia [17]. CIPN is an increasingly recognized dose-limiting toxicity. Unlike hematologic adverse effects, which can be managed with hematopoietic growth factors, there are no established prophylactic or specific therapeutic interventions for CIPN, and only symptomatic management is available [18]. The epidemiology of CIPN remains incompletely understood, but the condition predominantly presents as a sensory, length-dependent neuropathy, typically developing after cumulative drug exposure [18]. In most cases, the severity of CIPN is dose-dependent; however, for at least two agents (oxaliplatin and taxanes), immediate neurotoxic effects have also been observed [18].

Peripheral neuropathy associated with paclitaxel has become a critical factor in determining treatment tolerance and continuation. If grade 2 sensory peripheral neuropathy occurs (moderate symptoms that limit instrumental activities of daily living), according to the classification of the NCI-CTCAE version 5.0 [7], paclitaxel administration should be withheld until toxicity resolves or improves to grade 1, with a recommended dose reduction of 10 mg/m² [19]. In cases of grade 3 sensory peripheral neuropathy (severe symptoms that limit self-care activities of daily living), paclitaxel administration should be discontinued [19]. In our case, given the diagnosis of locally advanced breast carcinoma, a joint decision was made with the patient to pursue the most appropriate treatment strategy based on her clinical staging. This involved neoadjuvant chemotherapy with an anthracycline- and taxane-based regimen. As described, and always within a shared decision-making approach, a regimen of 12 weekly cycles of paclitaxel (80 mg/m²) was chosen, initially at a 50% dose reduction, followed by dose escalation in subsequent cycles, depending on treatment tolerance, particularly regarding peripheral neuropathy.

During the first five cycles, the patient demonstrated good treatment tolerance, allowing for dose escalation up to a 20% reduction from the full dose. However, after the fifth cycle, she experienced worsening peripheral neuropathy symptoms, leading to the decision to discontinue neoadjuvant chemotherapy after the eighth cycle of paclitaxel due to unacceptable toxicity, characterized by grade 3 peripheral neuropathy (according to the classification of the NCI-CTCAE version 5.0 [7]). This suggests that the cumulative dose of continuous paclitaxel administration may have a greater impact on peripheral neuropathy-related toxicity than the individualized dose per cycle.

Targeted therapy with CDK4/6 inhibitors in addition to endocrine therapy has been widely studied in early breast cancer. In the monarchE trial [20], the addition of abemaciclib for two years reduced the absolute risk of recurrence at four years by 6.4% (hazard ratio: 0.664, 95% CI: 0.578-0.762, p<0.0001) in a cohort of women with HR-positive, HER2-negative breast cancer who had either ≥4 involved LNs or one to three positive nodes with at least one of the following high-risk features: T3 tumors (greater than 5 cm of size), grade 3 histology, or Ki-67 expression ≥20%. The NATALEE trial [8] evaluated the addition of ribociclib (400 mg/day, administered on days 1-21 of each 28-day cycle) for three years to adjuvant ET in women with HR-positive, HER2-negative breast cancer, classified as stage II (either N0 with grade 2/3 histology and/or Ki-67 ≥20% or N1) or stage III, according to the eighth edition of the AJCC staging system [6]. The trial met its primary endpoint, demonstrating a 3.3% improvement in three-year invasive disease-free survival (hazard ratio: 0.748, 95% CI: 0.618-0.906, p=0.0014).

This case involves locally advanced HR-positive, HER2-negative breast cancer with a pathological staging of ypT2N2a, according to the eighth edition of the AJCC staging system [6], which confers a high risk of recurrence. Given that the patient did not complete the full recommended neoadjuvant chemotherapy regimen, a shared decision was made to initiate adjuvant endocrine therapy in combination with a CDK4/6 inhibitor.

In this case, due to the impossibility of administering abemaciclib, the decision was made to start ribociclib, taking into account the already published results of the NATALEE trial, despite the lack of formal indication for its use in this stage of breast cancer at the time. The patient only experienced grade 1 toxicities, according to the classification of the NCI-CTCAE version 5.0 [7]; however, due to clinical deterioration caused by an infectious complication leading to severe respiratory failure, it was decided to discontinue ribociclib after five months of treatment, while maintaining endocrine therapy as monotherapy. The decision to use ribociclib as an adjuvant therapy was due to the inability to use abemaciclib. While the combination of ribociclib and letrozole initially showed promise, a severe infectious complication resulting in respiratory failure led to treatment discontinuation, as the patient was no longer clinically fit to continue therapy. This emphasizes the critical need for close monitoring during treatment.

This report draws attention to the challenges of managing patients with neuromuscular comorbidities undergoing potentially neurotoxic oncological treatments. Peripheral neuropathy is a well-documented adverse effect of paclitaxel and often leads to dose reduction or treatment discontinuation, as occurred with this patient. Additionally, personalized management was essential to balance treatment efficacy against the risks posed by comorbidities. This report highlights the importance of a multidisciplinary approach in clinical decision-making, with active patient involvement, particularly in complex cases such as this.

## Conclusions

Pre-existing neuromuscular disorders, such as CMT disease, significantly increase the risk of CIPN and require careful planning of systemic therapy. Paclitaxel-related peripheral neuropathy may become dose-limiting even with initial dose reductions, underscoring the importance of close monitoring and individualized dose adjustments. Early identification and management of chemotherapy-related adverse effects are essential to preserve functional status and maintain treatment adherence in patients with comorbidities. CDK4/6 inhibitors, such as ribociclib, may be considered as adjuvant therapy in selected high-risk HR-positive breast cancer patients who are unable to complete standard chemotherapy, although off-label use must be carefully evaluated. Clinical decision-making in complex patients should involve a multidisciplinary approach and shared decisions, particularly when standard treatment options carry increased toxicity risks. Future research should focus on strategies to prevent and mitigate CIPN in high-risk populations, especially those with underlying peripheral neuropathies, to optimize oncologic outcomes without compromising quality of life.
